# Supplementary material for: Enrichment of Brain n-3 Docosapentaenoic Acid (DPA) and Retinal n-3 Eicosapentaenoic Acid (EPA) in Lambs Fed Nannochloropsis oceanica Microalga
Source: Animals (Basel). 2023 Feb 24;13(5):828. doi: 10.3390/ani13050828 (PMC10000189; doi:10.3390/ani13050828)
Supplement: Supplementary file 1 [file animals-13-00828-s001.zip › animals-2203429-supplementary.pdf]

Supplementary Material

Table S1. Summary table for total fatty acid (TFA) and dimethyl acetal (DMA) (mg/ g DM) content and composition (%TFA+DMA) of the liver tissues of lambs.

FA and DMA[1]	Diet[2]				SEM[3]	P-value
	C	O	SD	FD		
**TFA+DMA**	**80**	75	67	71	3.99	0.174
14:0	0.53	0.46	0.52	0.42	0.050	0.312
16:0	15.8	13.8	14.4	14.2	0.561	0.080
*c*9-16:1	1.17	1.07	1.19	1.31	0.151	0.715
17:0	1.56[b]	1.68[b]	1.73[b]	2.32[a]	0.195	0.039
18:0	24.7	24.8	23.5	24.0	0.946	0.764
*c*9-18:1	18.33[a]	11.48[b]	9.95[bc]	8.11[c]	0.730	<0.001
*c*11-18:1	2.07	1.95	1.90	2.50	0.298	0.462
18:2n-6	8.06	7.85	7.40	7.21	0.621	0.740
22:0	0.15	0.07	0.08	0.13	0.038	0.318
20:4n-6	7.88[a]	5.77[b]	7.01[a]	7.27[a]	0.407	0.007
20:5n-3	0.87[b]	4.25[a]	4.14[a]	4.68[a]	0.387	<0.001
22:5n-3	2.87[b]	8.18[a]	8.95[a]	9.19[a]	0.478	<0.001
22:6n-3	2.14[b]	4.19[a]	4.08[a]	2.88[b]	0.318	<0.001
**Partial sums**						
TC18[4]	59.9[a]	54[b]	52.1[b]	50.9[b]	1.28	<0.001
SFA[5]	43.2	41.3	40.8	41.7	1.13	0.480
MUFA[6]	28.3[a]	21.4[b]	21.4[b]	20.0[b]	1.12	<0.001
cis-MUFA	23.8[a]	15.7[b]	14.5[b]	13.0[b]	1.06	<0.001
PUFA[7]	26.9[b]	36[a]	36[a]	36.6[a]	1.83	0.002
n-3 PUFA	6.40[b]	17.5[a]	18[a]	17.7[a]	0.894	<0.001
n-6 PUFA	18.3	15.8	16.2	16.5	0.88	0.207
EPA + DHA	3.01[b]	8.45[a]	8.21[a]	7.56[a]	0.573	<0.001

Means within a row with different letters are significantly different ($P < 0.05$). [1]FA and DMA- fatty acids and dimethyl acetals. [2]C, control diet with no EPA sources; O, diet with *Nannochloropsis* sp. oil; SD, diet with spray-dried *Nannochloropsis oceanica* biomass; FD, diet with freeze-dried *Nannochloropsis oceanica* biomass. [3]Standard error of the mean; the value presented corresponds to a pooled sample standard error of the mean. [4]Sum of C18 FA.[5]Sum of saturated fatty acids. [6]Sum of monounsaturated FA. [7]Sum of polyunsaturated FA. In the FA notation (x:n-), '#' represents the number of C atoms, ':' the number of double bonds and 'n-' the location, in its carbon chain, of the double bond which is closest to the methyl end of the molecule. *c* stands for *cis*.
